# Better Predictions of Vitamin A Total Body Stores by the Retinol Isotope Dilution Method Are Possible with Deeper Understanding of the Mathematics and by Applying Compartmental Modeling

**DOI:** 10.1093/jn/nxz321

**Published:** 2019-12-18

**Authors:** Michael H Green, Joanne Balmer Green, Jennifer Lynn Ford

**Affiliations:** Department of Nutritional Sciences, College of Health and Human Development, The Pennsylvania State University, University Park, PA, USA

**Keywords:** model-based compartmental analysis, retinol isotope dilution, vitamin A stores, WinSAAM, stable isotopes, tracer kinetics

## Abstract

Retinol isotope dilution (RID) is a well-accepted technique for assessing vitamin A status [i.e., total body stores (TBS)]. Here, in an effort to increase understanding of the method, we briefly review RID equations and discuss their included variables and their coefficients (i.e., assumptions that account for the efficiency of absorption of an orally administered tracer dose of vitamin A, mixing of the dose with endogenous vitamin A, and loss due to utilization). Then, we focus on contributions of another technique, model-based compartmental analysis and especially the “super-person” approach, that advance the RID method. Specifically, we explain how adding this modeling component, which involves taking 1 additional blood sample from each subject, provides population-specific estimates for the RID coefficients that can be used in the equation instead of values derived from the literature; using model-derived RID coefficients results in improved confidence in predictions of TBS for both a group and its individuals. We note that work is still needed to identify the optimal time for applying RID in different groups and to quantify vitamin A absorption efficiency. Finally, we mention other contributions of modeling, including the use of theoretical data to verify the accuracy of RID predictions and the additional knowledge that model-based compartmental analysis provides about whole-body vitamin A kinetics.

## Introduction

Since Furr et al. (1) published the “Olson equation” in 1989, retinol isotope dilution (RID) equations have been used to estimate vitamin A total body stores (TBS) in humans (2). Although the technique is widely regarded as the best-available indirect method for assessing vitamin A status over a wide range (2), it has a number of limitations. For example, RID prediction equations include assumptions (coefficients); results cannot generally or confidently be verified by direct measurement; the optimal sampling time for application of the equation as a function of life stage or other variables (especially vitamin A stores and fractional catabolic rate) has not been established; and the technique has been assumed to be better at predicting TBS in groups than individuals. There is active interest in addressing these limitations since investigators need a feasible and reliable method for assessing vitamin A status.

In this article, we delve deeper into RID equations and their included assumptions, with the aim of enhancing understanding of the complexities of the method. Then, we discuss how addition of a super-person modeling component leads to improved confidence in RID predictions by providing population-specific values for the coefficients. We also highlight other ways that model-based compartmental analysis contributes to RID and to our understanding of vitamin A metabolism.

## RID Equations

Isotope dilution was originally explored by Bausch and Rietz (3) to estimate vitamin A stores in animals and humans. The rationale behind the technique is that, after a dose of labeled vitamin A is administered orally and enough time has elapsed for the dose to mix with body vitamin A pools, the tracer-to-tracee ratio [TTR; i.e., plasma retinol specific activity (SA)] measured in a single plasma sample can be used to predict liver or total body vitamin A.

Olson's group further developed the isotope dilution approach and presented an equation that used deuterium:hydrogen measured in plasma retinol after ingestion of a large dose of deuterated vitamin A (45 mg; equivalent to ∼2 mo worth of vitamin A for an average adult) to predict liver vitamin A in generally healthy adult surgical patients (1). Predictions for the group of 10 agreed quite well with the mean estimate of liver vitamin A based on liver biopsy. The Olson equation states the following:

(1) $$\begin{equation*}
{\rm{TLR}} = F \times dose \times \left\{ {S \times a \times \left[ {\left( {{\rm{H}}:{\rm{D}}} \right) - 1} \right]} \right\}
\end{equation*}$$

where TLR is vitamin A total liver reserves, *F* is the fraction of a dose of orally administered deuterated vitamin A that is absorbed and retained, *dose* is the administered tracer dose, *S* is the ratio of retinol SA in plasma to that in liver after mixing of tracer and tracee, *a* is a correction for fraction of the dose absorbed and retained but subsequently lost via catabolism, H:D is the postmixing hydrogen-to-deuterium ratio (i.e., 1/TTR) in plasma retinol, and −1 was described as a correction for the contribution of the dose to vitamin A stores. Furr et al. (1) used coefficients based on previous studies in rats and humans [*F* = 0.50 (3), *a* = 0.90 at 21 d (4), and *S* = 0.65 (5)] and recommended applying Equation 1 at 21 d after dosing. The value for *F* was determined based on the fraction of a labeled dose retained in the liver of rats 3–4 d after dosing (3), and the value for *S* came from analysis of rat plasma and liver SA at 7 d postdose (5). Thus, Equation 1 specifically predicts liver reserves (TLR), not TBS. However, if values for the coefficients were derived to reflect vitamin A in all exchangeable body pools, then the equation predicts TBS. While TBS are presumably concentrated in the liver in well-nourished individuals, that is not the case when vitamin A status is low (6).

The Olson equation (1) has since been used by other investigators (see references 2 and 7) and, in addition, 2 other RID equations are in use: an isotope mass balance equation (8, 9) and a modification of the Olson equation (10, 11), which we focus on here. The modified equation was developed after retrospective application of model-based compartmental analysis to plasma [^13^C_10_]retinol kinetic data collected in healthy young adults followed for 14 d after ingestion of [^13^C_10_]retinyl acetate. The modified equation (10) states the following:

(2) $$\begin{equation*}
{\rm{TBS}} = Fa \times S \times \left( {1/{\rm{S}}{{\rm{A}}_{\rm{p}}}} \right)
\end{equation*}$$

where *Fa* is fraction of an oral dose of labeled vitamin A that is absorbed and present in the body's exchangeable vitamin A storage pools at the time of blood sampling, *S* is the ratio of retinol SA in plasma versus stores at the same time, and SA_p_, the measured variable, is plasma retinol SA at that time, expressed as the fraction of dose/µmol plasma retinol. The coefficients in Equation 2 are closely related to those in Equation 1 [and to those used in the mass balance equation (8)] but, rather than being derived from the literature, they are defined by a compartmental model (see below). The modified equation was developed for use at an earlier time (4 or 5 d, compared with 21 d for the Olson equation) and it provided good agreement with model-predicted TBS in individuals (10). Although developed using data for young adults, Equation 2 has also been used to estimate TBS in Mexican children (12) and, as noted in reference (13), it will be applied in children from 3 other lower-income countries.

It is informative to rewrite and expand Equation 2 to predict TBS, or the amount of tracee plus tracer in stores, for a specific subject:

(3) $$\begin{equation*}
{\rm{TB}}{{\rm{S}}_{\rm{i}}} = \left( {F{a_{{\rm{it}}}} \times {S_{{\rm{it}}}}} \right)/{\rm{S}}{{\rm{A}}_{{\rm{pit}}}} = \left[ {F{a_{{\rm{it}}}} \times \left( {{\rm{S}}{{\rm{A}}_{{\rm{pit}}}}/{\rm{S}}{{\rm{A}}_{{\rm{sit}}}}} \right)} \right]/{\rm{S}}{{\rm{A}}_{{\rm{pit}}}}
\end{equation*}$$

where *Fa*_it_ is the fraction of the dose absorbed and found in stores for individual *i* at time *t* and *S*_it_ is the ratio of retinol SA in plasma (SA_pit_) to that in stores (SA_sit_) for individual *i* at time *t*. Then:

(4) $$\begin{equation*}
{\rm{TB}}{{\rm{S}}_{\rm{i}}} = {F{a_{{\rm{it}}}}\ /{\rm{S}}{{\rm{A}}_{{\rm{sit}}}}} = F{a_{{\rm{it}}}}/\left( {F{a_{{\rm{it}}}}/{{\rm{M}}_{{\rm{si}}}}} \right) = {{\rm{M}}_{{\rm{si}}}}
\end{equation*}$$

where M_si_ is the mass of vitamin A in stores for individual *i*and M_si_ is assumed to be constant over the duration of the RID study.

In practice, mean values used for the coefficients to estimate TBS in an individual are derived (*d*) either from the literature (in the case of the Olson and isotope mass balance equations) or from a super-person compartmental model (in the case of the modified Olson equation; see below). TBS predicted for a given subject using derived coefficients are as follows:

(5) $$\begin{equation*}
{\rm{TB}}{{\rm{S}}_{{\rm{dit}}}} = \left( {F{a_{{\rm{dt}}}} \times {S_{{\rm{dt}}}}} \right)/{\rm{S}}{{\rm{A}}_{{\rm{pit}}}}
\end{equation*}$$

and the relationship between the correct TBS value for that individual and the one calculated using derived coefficients is

(6) $$\begin{eqnarray*}
{\rm{TB}}{{\rm{S}}_{{\rm{dit}}}}/{\rm{TB}}{{\rm{S}}_{\rm{i}}} &=& \left[ {\left( {F{a_{{\rm{dt}}}} \times {S_{{\rm{dt}}}}} \right)/{\rm{S}}{{\rm{A}}_{{\rm{pit}}}}} \right]/\left[ {\left( {F{a_{{\rm{it}}}} \times {S_{{\rm{it}}}}} \right)/{\rm{S}}{{\rm{A}}_{{\rm{pit}}}}} \right]\nonumber\\
&=& \left( {F{a_{{\rm{dt}}}} \times {S_{{\rm{dt}}}}} \right)/\left( {F{a_{{\rm{it}}}} \times {S_{{\rm{it}}}}} \right)
\end{eqnarray*}$$

Note that not only SA_p_ but also both *Fa* and *S* are time variant. The TBS ratio will be closest to 1 when the derived value for *FaS* is closest to the individual's value. However, because the *FaS*ratio in the second expression of Equation 6 varies with time, predictions of TBS_dit_ will also be different at various times (JL Ford, MH Green, 2018, unpublished results).

## RID Coefficients

As implied above, application of an RID equation involves adjusting the measured variable (SA_p_) to account for the facts that vitamin A absorption is not 100%, that all of the absorbed dose does not exchange with vitamin A in stores before utilization, and that, because of further vitamin A utilization, not all of the dose that reaches stores is still there at the time of blood sampling; also, to predict the mass of vitamin A in stores by RID, we need *S* (SA_pdt_/SA_sdt_) derived either from the literature or from modeling.

It may be helpful to look more closely at the RID coefficients as well as mention studies that have aimed to update the values suggested by Furr et al. (1). The composite factor *Fa* is the fraction of the dose absorbed that has mixed with and is present in stores at the time of sampling. That is, looking back at Equations 1 and 2, it is evident that TBS predictions by RID are directly proportional to the value used for vitamin A absorption efficiency; thus, it is critical that we use the best-available estimate for absorption. Since it is not feasible to directly measure this variable as part of typical RID studies, investigators have relied on estimates from the literature, including those determined by fecal balance. Based on 3 d cumulative fecal excretion of label after ingestion of isotopic vitamin A, Sivakumar and Reddy (14) estimated a mean (±SD) absorption of 99.2% ± 1.0% for 5 apparently normal children; Aklamati et al. (15) reported mean values of 83.8% ± 7.1% for 4 children who consumed a high-dose vitamin A supplement (210 μmol) and 76.5% ± 9.5% for 4 who consumed a smaller dose (17.5 µmol). In previous studies, Tanumihardjo's laboratory has assumed a value of 90% for absorption in healthy children (9, 16) and Green's group has used 75% for adults (17, 18) and 80% for children (12, 13). As noted previously (11, 13, 19), information on vitamin A absorption efficiency could be obtained by a dual-label plasma isotope ratio method (20). Another approach would be to collect frequent blood samples during the absorption phase (e.g., 1–12 h), analyze both retinol and retinyl esters in plasma over time, and use compartmental analysis to quantify absorption efficiency. By doing such studies in different groups with varying vitamin A status and dietary sources of vitamin A, we could determine the variability in absorption efficiency among individuals and under various conditions.

In addition to absorption efficiency, RID coefficients also need to account for tracer loss between the time of dosing and blood sampling. Along with fecal measurements, cumulative urinary excretion of label was determined over 4–6 d in Sivakumar and Reddy (14) and Aklamati et al. (15); in Sivakumar and Reddy (14), the authors concluded that 82.2% ± 4.4% of the dose was absorbed and retained in normal children; corresponding values in Aklamati et al. (15) were 76.3% ± 6.7% (low dose) and 71.1% ± 9.4% (high dose). Note that absorption was 57.6% ± 6.0% in children with infection (14), with most of the difference due to increased catabolism or increased kidney filtration/excretion. Based on these results for healthy children, 75% seems reasonable as a value for *F* in Equation 1 and more realistic than the 90% used for absorption and retention in the mass balance equation (9, 16). Although researchers use the term “absorbed and retained” for the coefficient *F*, it is important to note that “retained” here describes the portion of the absorbed dose that is delivered from plasma to stores (i.e., exchangeable extravascular pools) rather than directly to tissues for utilization before the tracer has mixed with vitamin A in stores. Thus, a better descriptor for *F* in Equation 1 might be “absorbed and mixed with stores.”

The coefficient *a* as used in the Olson and isotope mass balance equations (8) assumes exponential loss and a rate constant equal to the system fractional catabolic rate; these assumptions are only appropriate if the label is homogeneously mixed immediately following dose administration. Since the label is ingested orally, time for mixing of tracer and tracee is required before the assumed exponential loss is reached and, thus, *a* as currently applied overestimates tracer catabolism. The coefficient *a* could be accurately estimated by using a compartmental model and differential equations, similar to how *Fa* is determined for the modified Olson equation (see next section). Because tracer catabolism occurs continually, first following rapid uptake of label from plasma into tissues and later when tracer has mixed with endogenous vitamin A, it makes sense to use a composite term *Fa*, as in Equation 2.

With regard to *S*, the ratio of retinol SA in plasma to that in stores, Haskell et al. (21) reported a value of 0.8 (range: 0.4–1.2) calculated 9–11 d after a large dose of labeled vitamin A (∼32.5 µmol) was administered to Bangladeshi surgical patients (mean vitamin A stores: 100 µmol) who were presumably consuming adequate vitamin A during the mixing period. This value was subsequently used by Van Stuijvenberg et al. (9) for subjects with high stores who were consuming high amounts of vitamin A. For studies in children (13), we have found that a value of 0.8 at 10 d is low for individuals with high vitamin A intakes and stores but should be appropriate at 14 d when status is low to adequate; for children with high to excess vitamin A status, *S* at 14 d is likely closer to 1.

## Model-Based RID: Improving RID Predictions by Addition of a Modeling Component

Model-based compartmental analysis (22) is an alternative to RID for estimating TBS. In contrast to an RID study, in which 1 blood sample is collected after dose ingestion, for modeling experiments, serial plasma samples are obtained over time and analyzed using the Simulation, Analysis and Modeling software [WinSAAM; www.winsaam.org (23)]; parameters describing whole-body vitamin A kinetics and pool sizes, including TBS, are determined. If serial samples are obtained from individuals (e.g., in adults as in reference 24), modeling can be done using each subject's data; in children, when serial sampling may not be feasible, one can apply a super-child approach, in which just 2 samples are collected from each subject and modeling is done on a composite dataset (12, 13). In some super-child studies [e.g., (12)], sampling times are set and then 2 times are randomly assigned to each child; in cases when the super-child approach is combined with RID [e.g., (13)], all children are sampled at a common time (e.g., 4 d) for calculation of individual subject values for TBS and at one other time during the established sampling schedule. The validity of the super-child method for quantifying retinol kinetics and TBS has been demonstrated using theoretical children (25), but it should be applicable to subjects of any age or physiological status. Importantly, as detailed below, one can also use modeling to obtain the RID coefficients used in Equation 2. This approach provides values for the coefficients that are specific for the group being studied and which can then be used to predict TBS in individuals at any time after initial mixing of the dose with stores.

**FIGURE 1:**
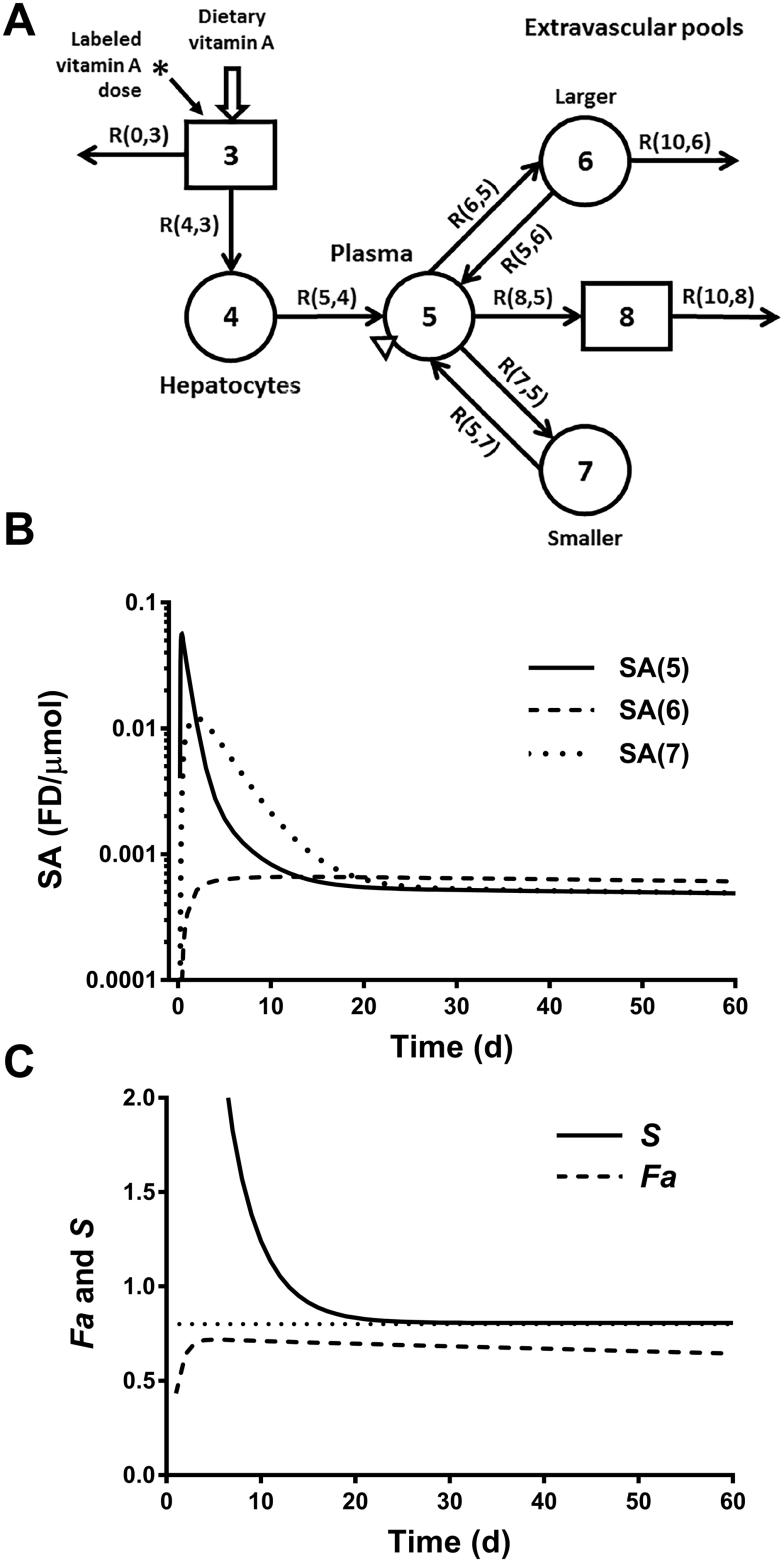
Compartmental model for vitamin A kinetics (A) used to simulate vitamin A specific activity in plasma and in stores (B) as well as for the retinol isotope dilution coefficients *Fa* and *S* (C). In the model [modified from (13)], circles represent compartments and rectangles represent delay components; arrows show transfer rates [R(I, J)s, or µmol of retinol transferred from compartment J to compartment I each day]; the asterisk shows the site of input of the labeled dose and the triangle shows the site of sampling. Among the compartments, compartment 5 represents plasma retinol; this exchanges with vitamin A in 2 extravascular storage compartments (the larger compartment 6 and the smaller compartment 7, which together represent vitamin A total body stores); system output is from compartment 6 and component 8. Panel B shows retinol SA simulated over time in compartments 5, 6, and 7 for a geometric mean dataset based on a study in Guatemalan children with relatively high vitamin A intakes (∼650 µg/d) (13). The curves in panel B can be interpreted in light of the underlying metabolism: thus, the time course for plasma shows that, after absorption and chylomicron processing in component 3 and compartment 4, labeled retinol in plasma (compartment 5) can be rapidly catabolized in extravascular tissues (component 8) or exchanged with vitamin A stores (compartments 6 and 7); unlabeled dietary vitamin A continues to enter the system. These processes lead to the drop in plasma retinol SA evident after ∼12 h. With time, retinol SA increases in stores and, since the majority of retinol input into plasma is from stores rather than diet (26), the drop in plasma SA begins to slow as tracer recycles from compartments 6 and 7, resulting in the bend in the curve at ∼4 d. Eventually, at ∼12 d, retinol SA in stores becomes greater than that in plasma. Panel C shows the retinol isotope dilution coefficients *Fa* (calculated as fraction of dose in compartments 6 plus 7) and *S* (calculated as SA in compartment 5/SA in compartments 6 plus 7) simulated over time for the same dataset; the dotted line indicates an equilibrium value for *S* of 0.8. For this group of subjects, the values for *Fa* were 0.72, 0.72, 0.70, and 0.70 at 4, 7, 14, and 21 d, respectively, and those for *S* were 4.1, 1.8, 0.95, and 0.83. FD, fraction of dose; SA, specific activity.

The compartmental model shown in **Figure 1**A (13, 26) is useful for illustrating how the RID coefficients are obtained from modeling; this model was adapted from that used in recently published super-child experiments (13). As indicated in the figure and further described in the legend, compartment 5 represents the plasma retinol pool and compartments 6 and 7 represent vitamin A TBS. Although only plasma is sampled, tracer response as the fraction of the dose or SA over time (Figure 1B) can be simulated in all 3 compartments using WinSAAM, reflecting the underlying metabolism. These simulations are used to calculate the RID coefficients included in Equation 2 [see Ford et al. (13, 25) for more details related to a geometric mean (super-child) model]. Thus,

(7) $$\begin{equation*}
F{a_{{\rm{dt}}}} = {\left[ {{\rm{F}}\left( 6 \right) + {\rm{F}}\left( 7 \right)} \right]_{\rm{t}}}\\
\nonumber
\end{equation*}$$

(8) $$\begin{equation*}
{S_{{\rm{dt}}}} = \left\{ {\left[ {{\rm{F}}{{\left( 5 \right)}_{\rm{t}}}/{\rm{M}}\left( 5 \right)} \right]} \right./\left\{ {{{\left[ {{\rm{F}}\left( 6 \right) + {\rm{F}}\left( 7 \right)} \right]}_{\rm{t}}}/\left[ {{\rm{M}}\left( 6 \right) + {\rm{M}}\left( 7 \right)} \right]} \right\}\\
\nonumber
\end{equation*}$$

(9) $$\begin{equation*}
{\left[ {FaS} \right]_{{\rm{dt}}}} = \left[ {{\rm{F}}{{\left( 5 \right)}_{\rm{t}}}/{\rm{M}}\left( 5 \right)} \right]/\left[ {{\rm{M}}\left( 6 \right) + {\rm{M}}\left( 7 \right)} \right],\\
\nonumber
\end{equation*}$$

where, at blood sampling time *t, Fa*_dt_ is the fraction of the dose that was absorbed and is still present in stores, F(I)_t_ is the fraction of the labeled dose in compartment I, *S*_dt_ is the ratio of retinol SA in plasma to that in stores, [*FaS*]_dt_ is the composite RID coefficient, and M(I) is the mass of vitamin A in compartment I; note that values for F(I) and M(I) are provided as part of the model solution. Changes in the coefficients *Fa* and *S* as a function of time are shown in Figure 1C. For *Fa*, the value peaks at 90% of the absorbed dose at 5 d and then slowly and gradually decreases as catabolism of tracer continues. For *S*, the value falls to and stays below 1 once SA_p_ crosses over SA in stores (SA_s_); 1 mo or so after dose ingestion, *S* will reach and remain at an equilibrium value (*S*_eq_) if dietary input of vitamin A is relatively constant. Through theoretical work, we have determined that the value for *S*_eq_ is R(5,6)/[R(5,4) + R(5,6)], where R(I, J) (Figure 1A) is the rate of vitamin A transfer to compartment I from compartment J. As an example, if input into plasma from diet [R(5,4)] is 25% of the input from stores [R(5,6)], then *S*_eq_ = 0.8, the value shown in Figure 1C. The higher the dietary intake of vitamin A, the farther below 1 will be the equilibrium value for *S*. If subjects are not consuming vitamin A, then *S* plateaus at a value of 1 since SA_p_ equals SA_s_ at isotopic equilibrium (27).

When modeling is done on a composite dataset, one obtains group mean values for the composite RID coefficient [*FaS*]_dt_. This value can be used to calculate TBS for individuals using each subject's measured SA_p_ at time *t* ≥4 d. We have shown using data for theoretical subjects that this method provides excellent prediction of TBS for the group (values within 17% of the known value) and reasonably accurate values for individuals (values within 25% for ≥66% of subjects) (25). In a field study, if all children are sampled at a common time (e.g., 4 d), calculating the individual values allows investigators to look at the range and rank order of TBS for subjects in the group; they can also calculate another value for TBS for each child whose second sample was obtained after 4 d, using the population value for *FaS* at that later time. In contrast, when using any time-invariant coefficients derived from the literature in the Olson or isotope mass balance equations, TBS should only be estimated at the time specified for those coefficients.

Although it would be ideal if one could recommend an optimal time for applying RID in children and adults over a wide range of stores, that is not (yet) possible. Present knowledge suggests that, when TBS is low, RID should be done early (e.g., 4 or 7 d), but when stores are high, later (e.g., 14 or 21 d) is better. From modeling (10), we know that the best time is when the CV% for *FaS* is lowest (e.g., 10–20%). As studies are done in various settings, researchers can determine the CV for *FaS* at various times and thus recommend when to apply RID, perhaps identifying a universal value for the composite coefficient at the time when the CV% for the composite coefficient *FaS* is lowest.

## Concluding Comments

Overall, the capacity of modeling to provide more accurate values for the RID coefficients helps to justify the additional resources required (specifically, a potentially longer study with additional blood sampling and the need for modeling expertise). If investigators are planning to do a traditional RID experiment in children, it is worth considering the addition of a super-child design, which requires just 1 additional blood sample from each subject, an estimate of vitamin A intake, and a duration of 28–42 d. This will yield not only population-specific values for the RID coefficients, thus improving confidence in the group mean RID estimate of TBS, but it will also allow for reasonably good predictions of TBS in individuals (25). Importantly, mean RID predictions of TBS can be compared to the model-predicted group estimate; expected agreement between the 2 estimates will increase confidence in both methods. By including modeling, one also obtains a wealth of information on whole-body vitamin A kinetics, adding to our understanding of vitamin A metabolism in different groups.

Finally, while an RID equation can readily yield a value for TBS, it is impossible to know whether that prediction is accurate—the need for a “gold standard” was highlighted by Quadro (28). In several recent publications (25–27, 29), Green's laboratory has addressed this need by using theoretical subjects for whom the kinetics and variables being studied (e.g., TBS) are known. One can then evaluate the method being tested (e.g., an RID equation) by comparing predictions to the known value. As additional advances are made in modeling the vitamin A system (13, 29), it is expected that the RID method will be even further improved.

## Notes

Financial support for this work was provided by the Bill & Melinda Gates Foundation (project number OPP1115464).

Author disclosures: The authors report no conflicts of interest.

Abbreviations used: RID, retinol isotope dilution; SA, specific activity; TBS, total body stores; TTR, tracer-to-tracee ratio; WinSAAM, Windows version of the Simulation, Analysis and Modeling software.
